# Treatment Response to SGLT2 Inhibitors: From Clinical Characteristics to Genetic Variations

**DOI:** 10.3390/ijms22189800

**Published:** 2021-09-10

**Authors:** Jasna Klen, Vita Dolžan

**Affiliations:** 1 Division of Surgery, Department of Abdominal Surgery, University Medical Centre Ljubljana, 1000 Ljubljana, Slovenia; jasna.klen@kclj.si; 2 Pharmacogenetics Laboratory, Institute of Biochemistry and Molecular Genetics, Faculty of Medicine, University of Ljubljana, 1000 Ljubljana, Slovenia

**Keywords:** SGLT2 inhibitors, cardiovascular safety, renal safety, genetic polymorphisms

## Abstract

SGLT2 (sodium-glucose cotransporter 2) inhibitors are a new class of antihyperglycaemic drugs that act on the proximal tubules of the kidney. They have shown efficacy in the management of diabetes mellitus type 2 and their cardiovascular and renal safety have been extensively investigated and confirmed in clinical trials. However, inter-individual differences in response to treatment with SGLT2 inhibitors may present in everyday clinical practice, and good predictors of glycemic response and the risk for adverse events in an individual patient are lacking. As genetic variability of SGLT2 may influence the treatment response, pharmacogenetic information could support the choice of the most beneficial treatment strategy in an individual patient. This review focuses on the clinical and genetic factors that may influence the treatment response to SGLT2 inhibitors in type 2 diabetes patients with comorbid conditions.

## 1. Introduction

Type 2 diabetes mellitus (T2DM) is a chronic, metabolic and progressive disease. The goal of treatment is good glycemic control, assessed by the hemoglobin A1C measurement, continuous glucose monitoring (CGM), and self-monitoring of blood glucose (SMBG). Evidence supports that in the long run, good glycemic control without large fluctuations in blood glucose levels prevents or delays microvascular complications such as diabetic nephropathy, neuropathy, and retinopathy. However, there are less data on the benefit of glycemic control in reducing macrovascular complications such as coronary artery disease, peripheral arterial occlusive disease (PAOD), and ischemic stroke [1].

The American Diabetes Association (ADA) provides comprehensive and evidence-based recommendations for the diagnosis and treatment of T2DM in their regularly revised and updated “Standards of Medical Care in Diabetes” [2]. These guidelines recommend metformin as the preferred initial pharmacologic agent in T2DM. Metformin has been in clinical use for more than 60 years and its mechanism of action is well known. It has pleiotropic effects, of which the inhibition of gluconeogenesis in the liver and the facilitation of glucose uptake into peripheral tissues contribute the most to glycemic control [3]. In recent years, several new agents, such as glucagon-like peptide 1 (GLP1) analogs, dipeptidyl peptidase-4 (DPP4) inhibitors, and selective sodium-glucose cotransporter 2 (SGLT2) inhibitors, were introduced for T2DM treatment, mostly as an add-on to first-line treatment.

SGLT2 (sodium-glucose cotransporter 2) inhibitors are a new class of insulin-independent anti-hyperglycemic drugs that inhibit glucose reabsorption in proximal tubules and, thus, affect glucose homeostasis via the kidneys [1]. In addition to other hormonal and signaling pathways that regulate glucose metabolism, the kidneys also play an important role in glucose homeostasis. SGLTs catalyze the active transport of glucose against concentration gradient across the apical (luminal) membrane by coupling it with the transport of sodium [4,5]. There are two SGLT isoforms; however, SGLT2 is the major isoform expressed in the first segment (S1) of the proximal tubules in the kidney and has a high capacity, but a poor affinity, for glucose. SGLT2 is also expressed in human pancreatic α-cells and regulates glucagon release [6]. The other isoform, SGLT1, has a high affinity, but a low capacity, for glucose. Although SGLT1 may be expressed in the kidney, it is mainly expressed in the gastrointestinal tract where it participates in the absorption of dietary glucose, and also in the liver [4,7,8].

The maximum capacity of kidney glucose reabsorption is 375 mg/min. Around 180 g of glucose is pre-filtered through the kidneys daily in subjects with normal glucose tolerance, so most of the glucose that is filtered in the primary urine in the glomeruli is reabsorbed back into the blood in the proximal tubules via SGLT. In healthy subjects, glucose is excreted in the urine when the plasma glucose concentration exceeds 10 mmol/L. In patients with high plasma glucose levels due to poorly controlled T2DM, the filtered glucose load exceeds the maximum capacity for glucose reabsorption, resulting in glycosuria. Hyperglycemia may be reduced by a decrease in glucose reabsorption via SGLT2 in the proximal convoluted renal tubules of the kidney. In this way, SGLT2 inhibitors lower the renal threshold for glucose excretion and, consequently, cause glucosuria. In patients who receive SGLT2 inhibitors, the amount of glucose excreted depends on the level of hyperglycemia and the glomerular filtration rate (eGFR), and is approximately 80 g per day [9].

## 2. Pharmacokinetics and Pharmacodynamics of SGLT2 Inhibitors

**Figure 1:**
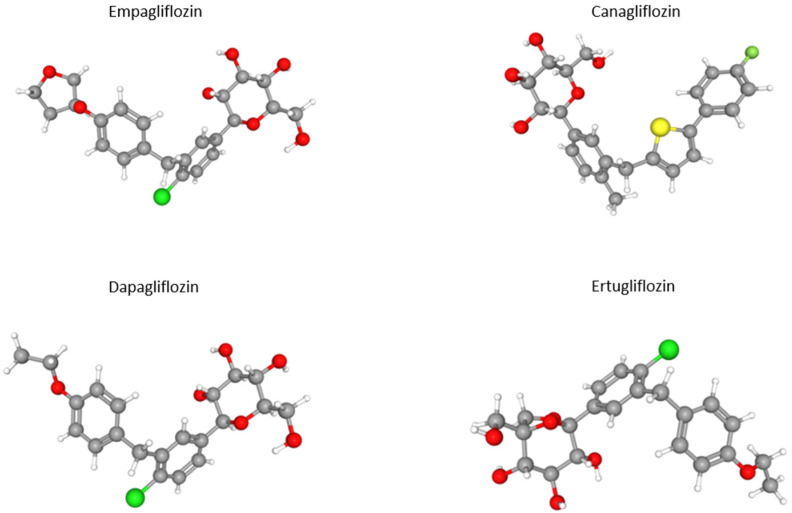
Three-dimensional structure of clinically used SGLT2 inhibitors [10]. Gray_carbon; red—oxygen; green—chloride; yellow—sulphur.

At present, four SGLT2 inhibitors are available on the market—dapagliflozin, empagliflozin, canagliflozin, and ertugliflozin (Figure 1).

Dapagliflozin (10 mg) was the first discovered highly potent SGLT2 inhibitor. The bioavailability of dapagliflozin is 78% and it is not altered by a high-fat diet, so the drug can be taken independently of food intake. It affects both fasting and postprandial plasma glucose levels. It is absorbed very rapidly, reaching peak plasma concentrations from one hour to one and a half hour after ingestion. The half-life (t_½_)- is 13 h, so it can be prescribed once a day. UGT1A9 enzyme is responsible for metabolism of dapagliflozin in the kidneys and liver. It is known that the dapagliflozin dose should be reduced to 5 mg in patients with hepatic impairment. Dapagliflozin is not recommended in patients with moderate and severe renal impairment or dialysis, nor in older patients. Dapagliflozin is mainly excreted in the urine [11,12].

Empagliflozin is most selective for SGLT2. It is taken once a day, regardless of food intake; the maximum daily dose is 25 mg per day. In total, 40% is excreted in the feces and 55% in the urine. Similar to other SGLT2 inhibitors, empagliflozin prolonged hepatic metabolism, predominantly by glucuronidation into inactive metabolites [11].

Canagliflozin is usually recommended before the first meal at a starting dose of 100 mg (especially in the elderly), which can be titrated to 300 mg. Its bioavailability is 65%. It is 99% protein bound. It reaches peak plasma concentrations after one to two hours. At a dose of 300 mg, the t_½_ is 13 h. Interactions with other drugs are not known. Use in patients with severe hepatic impairment is not recommended [11].

The most recent SGLT2 inhibitor on the market is ertugliflozin. In addition to empagliflozin, ertugliflozin has high selectivity for SGLT2. It is available as immediate-release tablets in doses of 5 and 15 mg. More than 85% of the total drug load is dissolved in 15 min and its t_½_ is 17 h. After one single dose, steady-state concentrations can be achieved by day 6. Its plasma protein binding is 93.6%. It is administered once daily as monotherapy or in combination with other antihyperglicemic drugs, regardless of meals. There is no need to adjust the dose in patients with renal impairment or mild-to-moderate hepatic impairment. Enzymes UGT1A9 and UGT2B are responsible for ertugliflozin metabolism [13].

## 3. Clinical Efficacy and Side Effects

SGLT2 inhibitors are clinically very effective. Numerous studies showed that they reduce the proportion of HbA1c by 0.80–1.03 in monotherapy and by 0.71–0.93 in combination with other antihyperglycemics. In addition to lowering plasma glucose levels, they also reduce body weight by 1.4–3.5 kg. It is well known that SGLT2 inhibitors may have an impact on LDL levels, leading to a modest or small increase, but the exact mechanism is still not clear. One hypothesis speculates that this effect could be associated with hemoconcentration due to natriuresis, and another suggests a decrease in LDL receptors’ expression on the surface of hepatocytes [14]. On the other hand, SGLT inhibitors may reduce levels of triglycerides, presumably due to improved insulin sensitivity as well as reduced glucotoxicity in β cell function, which decreases hepatic synthesis and increases the catabolism of triglyceride-rich lipoproteins [15]. SGLT2 inhibitors also affect uric acid levels as increased glycosuria may reduce urate absorption in the proximal convoluted tubule via GLUT9b. Due to the diuretic effect, they also lower blood pressure by 3–6 mm Hg [11].

SGLT2 inhibitors do not increase the risk of hypoglycemia. The most common side effect is increased susceptibility to mycotic infections resulting from glucosuria (more than 6.4% in women and 3–4% in men). Local antimycotic treatment is usually sufficient; there is no need to discontinue the drug. Nasopharyngitis may be more common with dapagliflozin [11].

Some clinical trials found associations between dapagliflozin and increased risk of bladder and breast cancer. Life-threatening diabetic ketoacidosis rarely develops in patients with T2DM, although it is necessary to be careful in patients with characteristic symptoms, even if they do not have high blood sugar levels [16].

The CANVAS program consisted of two studies, namely CANVAS (Canagliflozin Cardiovascular Assessment Study) and CANVAS R (Canagliflozin Cardiovascular Study-Renal), which examined cardiovascular, renal, and general safety in T2DM patients with a high risk of cardiovascular events. They found that in the canagliflozin group of patients with previous neuropathy, amputation, and peripheral vascular diseases, risk for amputation below the ankle increased 1.97-fold [17]. In canagliflozin users, in particular in older patients with lower eGFR and higher use of diuretics because of cardiovascular diseases, bone mineral density loss and increased risk for bone fracture were reported [18]. When using SGLT2 inhibitors in hypotensive patients, patients with renal diseases, or those on diuretics, especially loop diuretics, and the elderly, monitoring of body fluid volume and electrolyte concentration is recommended. The literature describes some cases of patients on SGLT2 inhibitors who developed Fournier’s gangrene [16,19].

## 4. Cardiovascular Complications and Safety

Cardiovascular diseases are the main cause of premature death in T2DM patients, in which mortality is two to four times higher compared to the general population. Hyperglycemia and insulin resistance decrease the bioavailability of nitric oxide and increase the accumulation of free radicals, which leads to endothelial dysfunction and increased inflammatory cytokines. All these conditions promote atherosclerosis. The prothrombotic environment also plays an important role. In T2DM, collagen synthesis and plaque stabilization are impaired due to reduced smooth muscle cell migration. Vascular remodeling is impaired, increasing the risk of ischemia and ulcers in patients with PAOD. In addition to the known independent risk factors for the development of cardiovascular disease, hyperglycemia and insulin resistance also lead to increased activity of the sympathetic and neurohumoral systems. Although it is generally accepted that good glycemic control reduces the risk of microvascular complications, there is no solid evidence regarding its effect on macrovascular complications. In patients with T2DM with cardiovascular disease and those at high risk for its development, SGLT2 inhibitors have a stimulating cardiovascular protective effect [20]. There are several theories about the mechanisms of cardiovascular protection of SGLT2 inhibitors. SGLT2 inhibitors increase sodium excretion and, thus, reduce intravascular volume, leading to a significant reduction in systolic blood pressure. In this way, the afterload is reduced and the oxygen consumption in the myocardium is reduced. SGLT2 inhibitors also affect neurohumoral pathways, in particular inhibition of the renin–angiotensin–aldosterone system. Mild hyperketonemia, which leads to increased absorption and oxidation of beta-hydroxybutyrate, helps to improve heart function. All of these mechanisms significantly reduce the risk of heart failure (HF) [20].

## 5. Renal Safety

Many cellular and molecular mechanisms are similar in cardiovascular and renal diseases. Sodium chloride causes *macula densa* to activate tubuloglomerular feedback and leads to the vasoconstriction of afferent arterioles, the reduction in glomerular hyperfiltration, and the normalization of intraglomerular pressure. Treatment with SGLT2 inhibitors is known to temporarily lower eGFR, but later slows the progression of chronic kidney disease and its complications [20]. SGLT2 inhibitors could lower albuminuria with mechanisms related to changes in hemodynamic function, renal hypoxia, and inflammation [21]. After treatment with ertugliflozin reduced eGFR, values returned to baseline and were higher after 104 weeks. In patients with albuminuria, ertugliflozin reduced the urine albumin/creatinine ratio (UACR), but the VERTIS-CV (eValuation of ERTUgliflozin Efficacy and Safety CardioVascular Outcomes) study did not observe significantly reduced composite renal endpoints compared to placebo [21,22].

CREDENCE (Canagliflozin and Renal Events in Diabetes with Established Nephropathy Clinical Evaluation) study examined renal outcome in patients with T2DM and pre-existing chronic kidney disease. Patients receiving an angiotensin converting enzyme (ACE) inhibitor or an angiotensin II inhibitor (ARB) were assigned to the placebo or canagliflozin group. The risk of renal failure and cardiovascular events was lower in the canagliflozin group [23].

Patients with an advanced renal disease with eGFR of 30 mL/min or more were included in the EMPA-REG (Empagliflozin Cardiovascular Outcome Event Trial in Type2 Diabetes Mellitus Patients-Removing Excess Glucose) study. Empagliflozin slowed the progression of renal failure, and there were fewer clinically relevant renal events in this group [24].

In the near future, we expect new data from the EMPA-KIDNEY (Study of Heart and Kidney Protection With Empagliflozin) study, which included approximately 6600 patients with chronic kidney disease who were treated with ACE inhibitors or ARBs [25]. Another ongoing study is the EMPEROR-Reduced study (Empagliflozin Outcome Trial in Patients with Chronic Heart Failure and a Reduced Ejection Fraction), which plans to enroll up to 3600 patients (with/without diabetes) with HF with reduced ejection fraction (HFrEF) [26].

## 6. Heart Failure and SGLT2 Inhibitors

It is known that patients with T2DM have an increased risk for HF with preserved ejection fraction (HFpEF) and HFrEF. Patients with T2DM have a significant prevalence of subclinical left ventricular (LV) diastolic dysfunction, which is an independent predictor of negative outcomes and a key cause of the development of HFpEF. Tissue hypoxia may further contribute to ventricular remodeling [27,28]. As heart failure progresses, renal failure also occurs, and this is associated with a poorer prognosis [29].

The DAPA-HF (Dapagliflozin And Prevention of Adverse-outcomes in Heart Failure) was a double-blind, placebo-controlled, event-driven study that included patients with HFrEF with and without type 2 diabetes on the optimal pharmacological therapy. No differences were found among different age groups, or between diuretic or, for example, sacubitril/valsartan users. In patients with HfrEF, one study found a minor impact on systolic blood pressure [30]. Regardless of baseline kidney function, dapagliflozin significantly reduced morbidity, mortality, and symptoms in patients with HFrEF when compared to placebo. The decline of kidney function was slower in the dapagliflozin group [31,32]. Dapagliflozin may thus present a new approach in the treatment of patients with HFrEF [33].

Ertugliflozin reduced the risk for first and total hospitalization due to outcomes in HF although SGLT2 inhibitors did not reduce hospitalizations due to atherosclerosis-related events [34]. The advantages of this class of drugs may be attributable to early beneficial hemodynamic effects on LV function rather than on atherosclerosis. The molecular mechanisms through which SGLT2 inhibitors lower hospitalizations due to HF are still unknown [27].

## 7. Cardiovascular Outcome Events and SGLT2 Inhibitors

The EMPA-REG study included patients with T2DM and with known cardiovascular disease and eGFR greater than 30 mL/min who received empagliflozin or placebo in addition to standard therapy. In the empagliflozin group, 10.5% of the 3-point major adverse cardiovascular events (3-point MACE: cardiovascular death, nonfatal myocardial infarction, or nonfatal stroke) were observed (RR in the empagliflozin group = 0.86; 95% CI = 0.74–0.99; *p* = 0.04 for superiority). The study showed statistically significant lower mortality due to cardiovascular events (38% relative risk reduction), less hospitalization due to heart failure (35% relative risk reduction), and death from any cause (32% relative risk reduction). A marked reduction in cardiovascular or all-cause mortality, hospital treatment for heart failure, and the onset or worsening of renal failure were observed in coronary bypass patients receiving empagliflozin. With these data, they proved the importance of secondary prevention of cardiovascular events after coronary bypass in this group of patients [35,36,37].

PAOD is one of the most common macrovascular complications and is a predictor of cardiovascular mortality. A subgroup of EMPA-REG included patients who underwent angioplasty, stenting, bypass surgery, lower limb amputation, or significant stenosis in one or more limbs and had an ankle index < 0.9 and ≥1. The primary endpoint was the same as in previous research. Patients receiving empagliflozin had a 43% reduction in cardiovascular mortality and a 38% reduction in all-cause mortality. There were also fewer 3-point and 4-point MACE (3-point MACE plus hospitalization for unstable angina) (16% and 7%, respectively). A high percentage (44%) also had less hospital treatment for heart failure. Acute renal failures or deterioration of chronic renal failure decreased by 46%. In the placebo group, 3.3% of patients with PAOD had major lower limb amputations. There were several minor amputations in the empagliflozin group. On the other hand, in patients without PAOD who received empagliflozin, only 0.9% had lower limb amputations, while 0.8% had minor amputations. The number of large amputations was the same in both groups [38].

The CANVAS program observed fewer primary events or mortalities in the canagliflozin group. The CANVAS study showed an increased risk of amputations, primarily at the level of the toes or feet, but the mechanism is not yet completely clear [39].

The DECLARE-TIMI 58 (Dapagliflozin Effect on Cardiovascular Events) study was multinational, randomized, double-blind, and placebo-controlled. In total, 17,160 patients with T2DM were included, of whom 10,186 were patients without atherosclerotic cardiovascular disease. Dapagliflozin did not reduce the number of MACE but was not inferior to placebo as it significantly reduced cardiovascular mortality or hospitalization due to heart failure. It also had a beneficial effect on kidney function [40].

VERTIS-CV was a multicenter, double-blind, randomized, placebo-controlled trial, which included T2DM patients, of which 75.9% had coronary artery disease, 22.9% cerebrovascular disease, 18.7% peripheral arterial disease and 23.7% had a history of heart failure. This is the highest percentage of patients with heart failure compared to the other three clinical trials of SGLT2 inhibitors. MACE occurred in 11.9% in the ertugliflozin group and the same in the placebo group. In total, 8.1% of ertugliflozin-treated T2DM patients and 9.1% patients on placebo were hospitalized for HF and cardiovascular death [22].

A summary of clinical trials investigating cardiovascular and renal outcomes of treatment with SGLT2 inhibitors is shown in Table 1. While empagliflozin and canagliflozin showed a decrease in MACE, dapagliflozin and ertugliflozin had a neutral effect. All four SGLT2 inhibitors, however, significantly reduced the number of hospitalizations due to heart failure.

## 8. Genetic Variability of SGLT2 Transporter in T2DM and Treatment with SGLT2 Inhibitors

SGLT2 is encoded by the *SGLT2* gene, also known as *SLC5A2* (solute carrier family 5 member 2), located on chromosome 16. Several mutations in the *SLC5A2* gene, affecting SGLT2 expression, membrane localization, or transporter function, were linked with familial renal glucosuria, characterized by abnormally high urinary glucose excretion in the presence of normal blood glucose levels [41,42,43]. In addition to these rare missense mutations, several common genetic variants were reported in the *SLC5A2* gene that could play a role in glucose homeostasis and could potentially influence the risk for T2DM as well as the response to treatment with SGLT2 inhibitors [5]. However, the findings that common *SLC5A2* genetic variants influence glucose homeostasis and metabolic traits in nondiabetic individuals, or that they are associated with the risk of T2DM, are not consistent among studies, as detailed below and in Table 2.

Enigk et al. investigated four intronic single nucleotide polymorphisms (SNPs) encompassing genetic variability within the *SGLT2* gene region and their association with the T2DM risk and related metabolic traits in two German cohorts. In the Sorb cohort that consisted of 1013 individuals, of which 106 had T2DM, 34 had impaired fasting glucose (IFG), 87 had impaired glucose tolerance (IGT), and 786 had normal glucose tolerance (NGT); none of the investigated SNPs showed any associations with the risk for T2DM. A lack of association of rs9934336 with the risk for T2DM was also observed in the validation cohort of 2042 individuals from the Metabolic Syndrome Berlin Potsdam Study that included 359 subjects with T2DM, 195 subjects with IFG, 329 subjects with IGT, and 1159 subjects with NGT. However, in 907 nondiabetic subjects from the Sorb cohort rs9934336, the AA genotype was associated with reduced glucose concentrations at 30 min and decreased insulin levels at 120 min during the oral glucose tolerance test (OGTT). In addition, rs3813008 was associated with insulin levels at 30 min, while rs3813007 was associated with glucose levels at 30 min during OGTT in the additive model. The combined analysis of both cohorts showed a nominal association of rs9934336 with insulin concentrations at 120 min during OGTT only in nondiabetic subjects [5].

These data suggested that some of the investigated variants could influence the proportion of glucose reabsorption by affecting baseline SGLT2 expression levels. Furthermore, it was proposed that such interindividual differences in SGLT2 expression levels might also influence the response to treatment with SGLT2 inhibitors, although SGLT2 inhibitors target this transporter directly. However, Zimdahl et al. performed a cross-sectional population study in a large cohort of 2600 metabolically well-phenotyped individuals at increased risk for T2DM and reported that, after correction for multiple testing, none of the five investigated common SNPs in the *SLC5A2* gene locus influenced diabetes-related metabolic traits such as body fat, insulin sensitivity/resistance, insulin release, HbA1c, plasma glucose, or systolic blood pressure. This cohort also included patients from four phase III trials of empagliflozin, with a total of 603 T2DM subjects receiving empagliflozin and 305 subjects receiving placebo. The investigated SNPs did not interfere with the response to empagliflozin treatment in T2DM patients and were not associated with HbA1c levels, fasting glucose, body mass, or systolic blood pressure in empagliflozin-treated patients [44].

As SGLT2 is also expressed in human pancreatic α-cells and SGLT2 inhibitors may elevate circulating glucagon concentrations, it was suggested that *SLC5A2* polymorphisms could modify circulating glucagon concentrations and hepatic glucose production. However, in a cohort of 375 healthy subjects at increased risk for T2DM, no associations were observed between these SNPs and plasma glucagon levels in the fasting state or upon glucose challenge with OGTT [6].

Three studies also investigated the associations between *SLC5A2* SNPs and late complications of T2DM. Drexel et al. genotyped a total of 1684 high-risk cardiovascular patients undergoing coronary angiography, among them 400 patients with T2DM, for three *SLC5A2* tagging SNPs (rs9934336, rs3813008, and rs3116150), to investigate their association with T2DM risk and cardiovascular complications. *SLC5A2* rs3813008 and rs3116150 were not associated with any glycemic parameters nor with T2DM, but rs9934336 was significantly associated with decreased HbA1c levels and decreased risk for T2DM. The protective effect of rs9934336 on T2DM risk was also confirmed by a meta-analysis that pooled their data with data from Enidgk et al. and Zimdhal et al., although individually, these two earlier studies failed to detect a significant association of this SNP with T2DM risk. On the other hand, the investigated SNPs were not associated with the risk for coronary artery disease (CAD) or the incidence of cardiovascular events in T2DM patients [45].

A study by Klen et al. that included 181 clinically well characterized Slovenian T2D patients observed a significant association between *SLC5A2* rs9934336 and increased fasting blood glucose levels as well as with aHbA1c levels under the dominant genetic model. After adjustment for T2D duration, a significantly higher risk for diabetic retinopathy was present in carriers of at least one polymorphic SLC5A2 rs9934336 A allele compared to non-carriers, but no associations were observed with the risk for other microvascular or macrovascular complications [46].

The most recent study by Katzmann et al. investigated associations between *SLC5A2* SNPs and the risk for heart failure to elucidate the mechanisms by which SGLT2 inhibitors reduce the risk of heart failure. In addition to 416,737 participants from the UK Biobank, they included a validation cohort of 3316 participants with high risk for cardiovascular events from the LUdwigshafen RIsk and Cardiovascular Health study (LURIC). The genetic score associated with lower risk of prevalent or incident heart failure in the UK Biobank included two intronic *SLC5A2* SNPs, s9934336, and rs3116150, both associated with the expression levels of the transporter. This association was also present in participants without T2DM or CAD and was mediated by several clinical factors. The associations of the genetic score with HbA1c, high-density lipoprotein cholesterol, uric acid, systolic blood pressure, waist circumference, and body composition mediated 35% of the effect of the genetic score on heart failure risk. This may suggest that, compared to the strong effect of pharmacologic SGLT2 inhibition, genetic variability may only have a modest effect. *SLC5A2* variants or genetic score were not associated with atherosclerotic cardiovascular disease outcomes either among participants from the UK Biobank or in the LURIC study [47].

## 9. Genetic Variability of Genes Coding for Drug Metabolizing Enzymes Involved in the Disposition of SGLT2 Inhibitors

Although most of the pharmacogenetic studies performed so far focused on *SLC5A2* gene coding as the major target of SGLT2 inhibitors, the pharmacokinetics of these drugs may be influenced by genetic variability in genes coding for drug metabolizing enzymes involved in their disposition. Glucuronidation reactions catalyzed by uridine diphosphate glucuronyltransferases (UGTs) are the most important mechanism that enables the elimination of inactive metabolites of SGLT2 inhibitors from the body via urine or feces. The main UGT involved in the disposition of SGLT2 inhibitors is UGT1A9; however, UGT2B4 and UGT2B7 were also shown to play a role. In vitro studies in liver microsomes and hepatocytes showed that hydroxylation and demethylation reactions by cytochromes P450 (CYP) may be involved in the Phase I metabolism of SGLT2 inhibitors in the liver [10].

Although glucuronidation plays a major role in the disposition of dapagliflozin and ertugliflozin, CYP1A1, CYP1A2, CYP2A6, CYP2C9, CYP2D6, and CYP3A4 were shown to be involved in the Phase I metabolism of both drugs [10]. On the other hand, the CYP3A4-mediated oxidative metabolism of canagliflozin was shown to be negliglible in humans [48]. Canagliflozin is, however, extensively metabolized by UGT1A9 and UGT2B4 into two inactive metabolites, M5 and M7, that are substrates of the efflux transporters ABCB1, ABCC2, and ABCG2 [49]. Empagliflozin undergoes minimal metabolism and, although it is metabolized to some extent via glucuronidation by UGT2B7, UGT1A3, UGT1A8, and UGT1A9, only approximately half of the parent drug is secreted as glucuronides in the urine, while, in feces, most of the parent drug can be found in the unchanged form [10].

Common functional polymorphisms in genes coding for these CYPs and UGTs were already shown to play a major role in the large interindividual variability in the pharmacokinetics, pharmacodynamics, and treatment response of several clinically important drugs [50]. For more than 100 gene–drug pairs, there is already a sufficient level of evidence that guidelines for personalized drug treatment tailored to an individual’s genetic makeup were prepared and published by professional societies such as the Clinical Pharmacogenetics Implementation Consortium [51,52] (CPIC), the Dutch Pharmacogenetics Working Group [53,54] (DPWG), and others.

However, no such evidence exists currently for SGLT2 inhibitors. There are no studies that have investigated the role of CYP and ABC transporter polymorphisms on the pharmacokinetics of SGLT2 inhibitors, and only one study so far investigated the effect of genetic variability of UGTs on canagliflozin pharmacokinetics in humans. For their pharmacogenetic analysis, Francke et al. have pooled 134 participants from 7 phase I canagliflozin studies, of which 5 included healthy subjects and 2 included T2DM patients. All the participants had available data on canagliflozin pharmacokinetics and were genotyped for common and potentially functional *UGT1A9* and *UGT2B4* polymorphisms. The study showed a significant impact of the UGT1A9*3 and UGT2B4*2 alleles on the steady-state pharmacokinetic parameters of canagliflozin and its two glucuronidated metabolites, M5 and M7. Canagliflozin plasma exposure was higher in UGT1A9*3 and UGT2B4*2 carriers than in non-carriers, and heterozygous UGT1A9*3 carriers had a larger increase in exposure than subjects homozygous for UGT2B4*2. However, in a population pharmacokinetic model, the levels of increased exposure were not considered to be clinicaly relevant and safety data from UGT1A9*3 carriers showed no apparent increase in the incidence of both overall adverse events as well as drug-related adverse events [55]. In addition, a larger population pharmacokinetic study that included 9061 pharmacokinetic samples from 1616 participants from nine phase I, two phase II, and three phase III studies showed no clinically relevant effect of UGT1A9*3 polymorphism on the pharmacokinetics of canagliflozin [56].

## 10. Conclusions

Large randomized clinical trials (RCT) have shown that the SGLT2 inhibitors currently used in everyday clinical practice effectively reduce cardiovascular morbidity and mortality. These trials provided evidence for the updated ADA/EASD guidelines for T2DM treatment, in which SGLT2 inhibitors have a central role. The latest ADA/EASD guidelines still recommend metformin as a first-line treatment. Additionally, in cases of already-known atherosclerotic cardiovascular disease, SGLT-2 inhibitors can be added in patients with eGFR above 60 mL/min. SGLT-2 inhibitors are always the first choice for add-on treatment in T2DM patients with heart failure. However, in patients with established cardiovascular disease and with several risk factors, the European Cardiovascular Society guidelines recommend the introduction of SGLT-2 inhibitors as first-line treatment, although, in most cases, RCT patients were treated with metformin in the first line [2]. Despite evidence that SGLT2 polymorphisms may play a role in glycemic control, more evidence on their impact on the outcomes of treatment with SGLT2 inhibitors is needed before genetic information might be used for the further personalization of T2DM treatment. Data on the role of genetic variability of drug metabolizing enzymes and drug transporters are still lacking; however, the current evidence does not support a major role of *UGT1A9* and *UGT2B4* polymorphisms in canagliflozin exposure and treatment safety, although glucuronidation plays a major role in the disposition of most SGLT2 inhibitors [55,56].

## Figures and Tables

**Table 1:** Clinical trials of cardiovascular and renal outcomes of treatment with SGLT2 inhibitors.

SGLT2 Inhibitors	Comparator	Study	No. of Patients	Median Follow Up (years)	MACE	Mortality		Heart Failure	Renal Outcome	Reference
						Cardiovascular	General			
Empagliflozin	Placebo	EMPA-REG	7020	3.1	↓	↓	↓	↓	↓	[35,36,37,38]
Dapagliflozin	Placebo	DECLARE-TIMI58	17,160	4.2	neutral	neutral	↓	↓	↓	[40]
Canagliflozin	Placebo	CANVAS	10,142	3.6	↓	↓	neutral	↓	↓	[39]
Canagliflozin	Placebo	CREDENCE	4200	2.6	NA	NA	NA	NA	↓	[23]
Ertugliflozin	Placebo	VERTIS-CV	8246	3.5	neutral	neutral	neutral	↓	↓	[22]

MACE—major adverse cardiovascular events; NA—not applicable.

**Table 2:** *SGLT2* genetic variability in T2DM and in treatment with SGLT2 inhibitors.

*SLC5A2* SNPs	Study Population	Outcome Studied	Main Findings	Reference
rs9934336 rs3813007 rs3813008 rs3116150	1013 subjects from German Sorb cohort: 106 with and 907 without T2DM; Validation: 2042 subjects from Metabolic Syndrome Berlin Potsdam Study: 359 with and 1683 without T2DM	T2DM risk, metabolic traits, glycemic control, and insulin levels after OGTT	No associations with T2DM risk; rs9934336 AA genotype associated with reduced glucose levels at 30 min and decreased insulin levels at 120 min of OGTT in nondiabetic subjects	[5]
rs3116149 rs9934336 rs3813008 rs11646054 rs3116650 rs9924771	2229 subjects from Tübingen Family (TÜF) study: 1558 glucose tolerant and 671 prediabetic; 603 T2DM subjects on empagliflozin and 305 on placebo	T2DM risk, metabolic traits, response to empagliflozin	No association with metabolic traits; No association with response to empagliflozin	[44]
rs9924771 rs3116150 rs3813008 rs9934336	375 subjects at increased risk for T2DM	Plasma glucagon concentrations in the fasting state and during OGTT	No association with plasma glucagon levels	[6]
rs9934336, rs3813008, and rs3116150	1684 subjects undergoing coronary angiography including 400 patients with T2DM Meta-analysis of data from 3 studies	T2DM risk, risk for CAD (coronary artery disease), incidence of cardiovascular events	rs9934336 associated with decreased HbA1c and decreased T2DM risk; No association with CAD or incidence of cardiovascular events; rs9934336 association with T2DM risk confirmed in a meta-analysis	[45]
rs9934336	181 Slovenian T2DM patients	Glycemic control, risk for macro or microvascular complications	rs9934336 associated with increased fasting blood glucose levels and HbA1c; Higher risk for diabetic retinopathy in polymorphic rs9934336 A allele carriers compared to non-carriers; No association with other micro or macrovascular complications	[46]
SNPs with MAF > 0.01: rs9934336 and rs3116150 included in SGLT2 genetic score	Data on 416,737 UK Biobank subjects; Validation: 3316 subjects from LUdwigshafen RIsk and Cardiovascular Health study (LURIC)	Heart failure risk	Nominal association of SGLT2 genetic score with reduced T2DM risk; SGLT2 genetic score associated with lower risk of prevalent or incident heart failure; No association with atherosclerotic cardiovascular disease outcomes or markers	[47]

T2DM—type 2 diabetes mellitus; SNPs—single nucleotide polymorphisms; OGTT—oral glucose tolerance test; CAD—coronary artery disease.
